# eOSCE stations live versus remote evaluation and scores variability

**DOI:** 10.1186/s12909-022-03919-1

**Published:** 2022-12-13

**Authors:** Donia Bouzid, Jimmy Mullaert, Aiham Ghazali, Valentine Marie Ferré, France Mentré, Cédric Lemogne, Philippe Ruszniewski, Albert Faye, Alexy Tran Dinh, Tristan Mirault, on behalf of Université Paris Cité Osce study group, Nathan Peiffer Smadja, Léonore Muller, Laure Falque Pierrotin, Michael Thy, Maksud Assadi, Sonia Yung, Christian de Tymowski, Quentin le Hingrat, Xavier Eyer, Paul Henri Wicky, Mehdi Oualha, Véronique Houdouin, Patricia Jabre, Dominique Vodovar, Marco Dioguardi Burgio, Noémie Zucman, Rosy Tsopra, Asmaa Tazi, Quentin Ressaire, Yann Nguyen, Muriel Girard, Adèle Frachon, François Depret, Anna Pellat, Adèle de Masson, Henri Azais, Nathalie de Castro, Caroline Jeantrelle, Nicolas Javaud, Alexandre Malmartel, Constance Jacquin de Margerie, Benjamin Chousterman, Ludovic Fournel, Mathilde Holleville, Stéphane Blanche

**Affiliations:** 1 Université Paris Cité and Université Sorbonne Paris Nord, Inserm IAME, F-75018 Paris, France; 2 Emergency Department, Bichat-Claude Bernard University Hospital AP-HP, Paris, France; 3 Virology laboratory, Bichat-Claude Bernard University Hospital AP-HP, Paris, France; 4 Département d’Épidémiologie, Biostatistique et Recherche Clinique, Bichat-Claude Bernard University Hospital AP-HP, Paris, France; 5 UFR de Médecine, Université Paris Cité, Paris, France; 6 Université Paris Cité, INSERM U1266, Institut de Psychiatrie et Neuroscience de Paris, F-75014 Paris, France; 7 Service de Psychiatrie de l’adulte, AP-HP, Hôpital Hôtel-Dieu, F-75004 Paris, France; 8 Service de gastro-entérologie et pancréatologie, Hôpital Beaujon AP-HP, Paris, France; 9 Service de Pédiatrie Générale, Hôpital Robert Debré AP-HP, Paris, France; 10 Département d’Anesthésie-Réanimation, Hôpital Bichat-Claude Bernard, AP-HP, Paris, France; 11 Département de médecine vasculaire, Hôpital Européen Georges Pompidou AP-HP, Paris, France; 12 Université Paris Cité, PARCC team 5, INSERM U970, F-75015 Paris, France

**Keywords:** Interrater reliability, Remote objective structured clinical examination, Global ratings

## Abstract

**Background:**

Objective structured clinical examinations (OSCEs) are known to be a fair evaluation method. These recent years, the use of online OSCEs (eOSCEs) has spread. This study aimed to compare remote versus live evaluation and assess the factors associated with score variability during eOSCEs.

**Methods:**

We conducted large-scale eOSCEs at the medical school of the Université de Paris Cité in June 2021 and recorded all the students’ performances, allowing a second evaluation. To assess the agreement in our context of multiple raters and students, we fitted a linear mixed model with student and rater as random effects and the score as an explained variable.

**Results:**

One hundred seventy observations were analyzed for the first station after quality control. We retained 192 and 110 observations for the statistical analysis of the two other stations. The median score and interquartile range were 60 out of 100 (IQR 50–70), 60 out of 100 (IQR 54–70), and 53 out of 100 (IQR 45–62) for the three stations. The score variance proportions explained by the rater (ICC rater) were 23.0, 16.8, and 32.8%, respectively. Of the 31 raters, 18 (58%) were male. Scores did not differ significantly according to the gender of the rater (*p* = 0.96, 0.10, and 0.26, respectively). The two evaluations showed no systematic difference in scores (*p* = 0.92, 0.053, and 0.38, respectively).

**Conclusion:**

Our study suggests that remote evaluation is as reliable as live evaluation for eOSCEs.

**Supplementary Information:**

The online version contains supplementary material available at 10.1186/s12909-022-03919-1.

## Background

Objective structured clinical examinations (OSCEs) are considered a fair evaluation method for health students since they aim to assess their competencies in a standardized and objective way [1]. Several factors have been established as influencing OSCE reliability, including the duration, circuit, sites, scoring system, and rater [2, 3], with extremely heterogeneous conclusions. In OSCEs with an extremely large number of students, the practice of conducting multiple parallel versions of the same examination with different raters can also introduce rater variability [4]. Therefore, each cohort of raters should evaluate performances with the same standard of judgment to ensure that students are not systematically either advantaged or disadvantaged by their circuit and guarantee the fairness of OSCEs. Few studies have examined the influence of different circuits on OSCE examinations [5–7]. Their findings were heterogeneous, probably because in most studies, students are fully nested within cohorts of examiners with no crossover between groups of students and groups of examiners, thus preventing assessing rater cohort variability. Yeates et al. developed a video-based method to adjust for examiner effect in fully nested OSCEs and showed that examiner cohorts could substantially influence the scores of students and could potentially influence the categorization of around 6.0% of them. One student (0.8%) passed who would otherwise have failed, whereas six students (5.2%) failed who would otherwise have passed [4].

The COVID-19 pandemic forced medical schools globally to cancel the on-site OSCEs [8–11]. To the best of our knowledge, data on examiner effects, score variance, and online OSCEs remain scarce. We exploited a large-scale online OSCE (eOSCE) at the Université Paris Cité medical school [12], allowing live and remote evaluation, to assess the agreement between live and remote video-based evaluations and quantify the score variability corresponding to student ability and the rater effect on the global score for the station and at the item level.

## Methods

### Study design

The medical school of the Université de Paris Cité conducted eOSCEs as a mock examination on June 2021, using the video conferencing platform Zoom; 531 students in their fifth year of medical school and 298 teachers participated.

We conducted a double evaluation on a sample of recorded students’ performances’ during the three eOSCE stations.

This study obtained the approval of the ethics committee of the Université Paris Cité, CER U-Paris N° 2021-96-BOUZID. The ethics committee of Université Paris Cité waived the need for written informed consent from the students but required that they received clear information about the study protocol with the possibility to decline to participate to the training.

### Population

Medical students completing their fifth year at the Université Paris Cité medical school (Paris, France) were invited to participate on a voluntary basis in the first large-scale eOSCE in our school. Teachers from the medical school of the Université Paris Cité with previous experience of on-site OSCEs administered the eOSCE and were involved as raters or standardized patients.

### Description of eOSCE station

We proposed a circuit of three eOSCE stations to the students. Expert teachers from the Université de Paris Cité OSCE group carefully prepared these stations. Each station was evaluated by two other teachers and previously tested with volunteer residents to assess its feasibility within allocated time. Station#1 concerned gynecology and focused on history-taking skills. Station#2 concerned addictology and evaluated communication and history-taking skills. Station#3 concerned pediatrics; it provided a picture of chickenpox’s lesions and considered therapeutic management strategy skills. None of these stations addressed any technical procedures or clinical examination skills to accommodate the digital environment and allow more straightforward remote evaluation.

Each station lasted 7 min, and the student was then invited to click on the next link for the following station. The scoring system was binary (Fulfilled/Not fulfilled) for each item, and the items were weighted differently.

### OSCE evaluation

The raters observed the OSCE station with both their camera and microphone turned off. They then completed the evaluation grid online on the Université Paris Cité usual software, “Sides THEIA.”

Four weeks after the eOSCEs, the videos were uploaded on a secured institutional online platform, and a panel of 35 volunteer raters watched 236 randomly selected stations, completing a double evaluation. They were able to pause and rewatch the videos as much as they wanted.

### Objectives

The primary objective was to compare the live online evaluation with the remote online evaluation of these eOSCEs.

The secondary objective was to assess the other score variability components: students and raters’ effect, raters’ experience, students’ genders, and the evaluated items.

### Statistical analysis

Separate descriptive analyses were performed for the three stations. We reported score dispersions, the success percentage for each item, and discrimination. Discrimination indicates how much better the best students perform than others for a specific item. This is defined as the difference in success rate (or score) between the subset of the 30% students with best performances and 30% students with worst performances. These subsets refer to the station’s score, whereas discrimination is computed for each item.

In our context of multiple raters (live and remote evaluation) and multiple students, we fitted a linear mixed model with student and rater as random effects and the score as an explained variable, allowing estimation of intraclass correlation coefficients (ICCs, also referred to as variance partition coefficients) for student and rater. Three linear models were fitted, one for each station. The rater ICC represents the variance of the score due to the rater, expressed as a proportion of the total variance (rater, student, and residual). A low rater ICC indicates a relatively homogeneous notation or, at least, a low effect of rater heterogeneity on score dispersion [13]. We also estimated the student ICC: a high student ICC indicates that the observed dispersion of the scores is almost entirely due to the dispersion of the student’s skills.

The influence of the gender and experience of the rater was tested by including fixed effects in the model, and we reported the corresponding Wald *p*-value. The experience was classified binarily; experienced raters were tenured academic physicians. The same strategy was used to test the influence of student gender and the timing of the evaluation: live or remote; *p*-values below 5% were considered statistically significant.

Each station comprised 18 to 28 items, for which the notation was binary, and we also investigated the sources of variability of scores at the item level. We fitted a mixed logistic model for each item to evaluate student and rater ICC at item level according to the latent variable approach described by Goldstein et al. [14]. We also reported crude agreement at an item level, defined as the number of students for which both raters agree, even if its interpretation can be misleading since part of this crude agreement is due to chance. For all models (linear and logistic), variance estimates were obtained based on the restricted maximum likelihood (REML) with the lme4 package of R 4.1.2 software. Missing values were not imputed, and the analysis was limited to available data.

## Results

A total of 202 students participated in at least one station; 131 (65%) were female. The first station comprised 18 separate items. After purging for missing data and removing students who were only evaluated once, 170 observations, corresponding to 85 students and nine raters, were analyzed. For the two other stations, using the same quality control, we retained 192 and 110 observations for the statistical analysis, corresponding to 96 and 55 students, and 15 and seven raters, respectively.

Of the 31 raters, 18 (58%) were male. Scores did not differ significantly according to the gender of the rater (*p* = 0.96, 0.10, and 0.26). There was also no systematic difference in scores according to the evaluation timing (live or remote, *p* = 0.92, 0.053, and 0.38). Twenty raters were experienced physicians, but no association was found between the rater’s experience and scores for Station#1 and Station#3 (*p* = 0.26 and 0.12, respectively). For Station#2, experienced raters gave higher scores (mean score difference 5.4, 95% CI 4.5–10.8, *p* = 0.048). The gender of the student was not associated with their score (*p* = 0.32, *p* = 0.57, and *p* = 0.25 for the three stations).

Table 1 summarizes the results of the different models. The median score (out of 100) and interquartile range were 60 (IQR 50–70), 60 (IQR 54–70), and 53 (IQR 45–62) for the three stations. The score variance proportions explained by the rater (namely, the rater’s ICC) were 23.0, 16.8, and 32.8%. Some items had an extremely high success rate and thus low discrimination. Item 10 of Station#3 (chickenpox diagnosis) was passed for all students, leading to a 100% success rate and 0% discrimination. Two items (one in Station#2 and another in Station#3 of medical history and therapeutic education) had negative discrimination.

**Table 1:** Summary of the factors influencing students ‘scores variability

	Station 1				Station 2				Station 3			
	Score median- IQR (Q1-Q3) or % success	Agreement N (%)	Students’ ICC (%)	Raters’ ICC (%)	Score med (Q1-Q3) or % success	Agreement N (%)	Students’ ICC (%)	Raters’ ICC (%)	Score med (Q1-Q3) or % success	Agreement *N* (%)	Students’ ICC (%)	Raters’ ICC (%)
**score (/100)**	60 (50-70)	21 (25)	60.2	23.0	60 (54-70)	19 (20)	59.9	16.8	53 (45-63)	7 (13)	39.4	32.8
item 1	65	76 (89)	97	3	61	88 (92)	*	*	52	54 (98)	*	*
item 2	93	75 (88)	82	18	64	90 (94)	*	*	84	49 (89)	*	*
item 3	94	74 (87)	*	*	99	95 (99)	*	*	47	41 (75)	57	8
item 4	95	76 (89)	*	*	39	79 (82)	77	2	55	48 (87)	84	3
item 5	33	67 (79)	31	48	38	90 (94)	99	1	61	44 (80)	71	1
item 6	48	80 (94)	92	0	79	89 (93)	*	*	72	46 (84)	84	16
item 7	58	69 (81)	72	7	15	83 (86)	87	13	45	42 (76)	62	2
item 8	75	72 (85)	89	11	71	79 (82)	93	6	6	52 (95)	*	*
item 9	64	79 (93)	99	0	15	94 (98)	*	*	56	47 (85)	82	0
item 10	28	80 (94)	*	*	86	84 (88)	*	*	100	55 (100)	*	*
item 11	85	76 (89)	93	7	57	66 (69)	58	15	33	43 (78)	74	7
item 12	74	70 (82)	*	*	73	88 (92)	*	*	26	30 (55)	1	75
item 13	28	76 (89)	*	*	59	88 (92)	99	0	82	43 (78)	47	1
item 14	71	72 (85)	97	1	98	92 (96)	*	*	49	43 (78)	57	10
item 15	30	78 (92)	98	2	16	86 (90)	*	*	55	45 (82)	71	14
item 16	25	72 (85)	97	0	93	88 (92)	65	35	29	47 (85)	96	3
item 17	39	64 (75)	61	10	36	72 (75)	39	44	97	52 (95)	*	*
item 18	53	73 (86)	*	*	93	86 (90)	84	14	57	46 (84)	*	*
item 19									29	47 (85)	97	1
item 20									35	50 (91)	97	2
item 21									40	43 (78)	66	20
item 22									63	42 (76)	*	*
item 23									7	49 (89)	*	*
item 24									45	46 (84)	71	8
item 25									54	44 (80)	69	2
item 26									95	52 (95)	71	29
item 27									49	47 (85)	66	19
item 28									85	40 (73)	9	20

Students’ Intraclass Correlation Coefficients (ICC) and Raters’ ICC at station and item level respectively

The mark * indicates that either the model failed to converge or a singular fit was obtained. Results are thus not reliable. Lowest rater ICC indicates a more homogenous notation

** Agreement is defined as the number of students for which both raters agree at an item level

The item-level analysis showed extremely high variability between items. Some items showed a high proportion of variance explained by the rater (e.g., in the first station, item 5 concerning medical history had an estimated rater ICC of 0.48). Conversely, most of the items showed a reasonable rater ICC. All agreement proportions appeared fair since only one was below 73%. Note that for an item with nearly complete agreement or a high proportion of success, the statistical model may fail to converge or return a singular fit, resulting in 22 items out of 64 not being analyzed.

## Discussion

To our knowledge, this study is the first to compare live and remote evaluations of eOSCEs. We found no significant difference between the live and remote evaluations. Previous studies showed that remote evaluation using a video recording system is as reliable as live in-person evaluation in on-site OSCEs [15, 16]. Our findings are consistent with the conclusion of Yeates et al. that internet-based scoring could potentially offer a more flexible means to facilitate scoring and minimize the examiners’ cohort effect [17]. Chen et al. even emphasized that on-site evaluation could introduce an audience effect that could influence the students’ performances [15]. One of the greatest challenges for OSCE organizers is to recruit available teachers for the evaluation. Remote evaluation might therefore enable fewer examiners to work simultaneously.

The score variance proportion explained by the rater was moderate for the three stations comprising our eOSCEs. The gender of our raters did not influence the scoring, but experienced raters scored higher than junior raters in Station#2. This finding contrasts with the findings of Chong et al. on the raters’ experience since they demonstrated that junior doctors scored consistently higher than senior doctors in all domains of OSCE assessment [18, 19]. However, Station#2 in our study, concerning alcohol addiction, had the lowest rater ICC and, therefore, the more homogenous evaluation between raters. More experienced raters scored higher than juniors. Regarding students’ ICC, they are slightly lower than those reported in previous publications on interrater-reliability in on-site OSCEs [20, 21]. Per instance, in this study by Hurley et al. which objective was to assess inter-observer reliability and observer accuracy as a function of OSCE checklist length. Inter-rater reliability ranged from 58 to 78% (corresponding to students’ ICC in our study that ranged from 39.4 to 60.2%) [22].

The item analysis showed a reasonable rater ICC with good agreement proportions. However, few items showed a high proportion of variance explained by the rater. In the 5th item of Station#1, regarding medical history and, more precisely, endometrial cancer risk factor research, the rater ICC was higher than in other items, suggesting that this item was not clearly explained in the scoring process.

Regarding the students’ profiles, this study showed no impact of the student’s gender on OSCE scores, also confirming the findings of Humphrey-Murto et al. in a study evaluating simulated patients’ gender and students’ gender on OSCE grading [23].

## Limitations

Our study has some limitations. First, OSCE stations mainly focused on communication and history-taking skills, so the video interface was suitable. Still, a recent review suggests that it may be helpful to employ multiple cameras for more technical tasks and rely on more advanced simulation methods. All agreement proportions were fair; however, this might be partly explained by chance, especially for items with a high success rate.

## Conclusion

Our study suggests that remote evaluation is as reliable as live evaluation for eOSCEs. It also, highlights that the score variance proportion explained by the rater is significant even with eOSCEs and that high variability exists between items. These data encourage us to continue improving the OSCE station writing process. Further studies are required to compare the variability of the scores between online and on-site OSCEs.

## Supplementary Information

**Additional file 1.**

## Data Availability

Data available on demand. Any request should be addressed to the corresponding author.
