# Supplementary material for: eOSCE stations live versus remote evaluation and scores variability
Source: BMC Med Educ. 2022 Dec 13;22:861. doi: 10.1186/s12909-022-03919-1 (PMC9745699; doi:10.1186/s12909-022-03919-1)
Supplement: Supplementary file 1 — Additional file 1. [file 12909_2022_3919_MOESM1_ESM.docx]

| Items | Coefficient |
| --- | --- |
| Says “Hello”, introduces himself, her, their selves “I am Doctor X” | 10 |
| Attitude towards the patient (condescending, courteous, polite, empathetic?) | 5 |
| Ability to listen | 5 |
| Ability to question | 5 |
| Asks for personal medical history including diabetes and overweight (give points if  research of these two risk factors) | 5 |
| Asks for personal surgical history | 5 |
| Asks for personal gynecological history: Parity and anamnesis of menopause (give points if he/she addresses both items) | 5 |
| Asks for personal gynecological history: history of breast cancer with tamoxifen intake | 5 |
| Asks for family history of gynecological and non-gynecological cancers  (Search for familial cancers suggestive of Lynch syndrome) | 10 |
| Asks for lifestyle habits: Tobacco, alcohol, diet | 5 |
| Asks about the patient's symptoms: the date of onset of symptoms specified | 5 |
| Clarifies heavy bleeding  (can be evaluated by the frequency of protection change) | 5 |
| Specify the origin of the bleeding: vaginal? urinary? digestive? | 5 |
| Asks about functional urinary signs | 5 |
| Asks about functional digestive signs | 5 |
| Asks about signs of anemia (fatigue, pallor, dyspnoea, etc.) | 5 |
| Asks about the deterioration of the general state: asthenia, weight loss, anorexia  (give points if at least two items were searched) | 5 |
|  |  |

STATION 2 evaluation grid

| Items | Coefficient |
| --- | --- |
| Says “Hello”, introduces himself, her, their selves | 0.5 |
| Presents him, her, their function | 0.5 |
| Evaluates alcohol consumption in glasses, units, or grams/day  or per week | 0.5 |
| Looks for signs of addiction  (At least one item among: morning consumption, tremors or  cravings relieved by drinking alcohol) | 0.5 |
| Search for a psychiatric history (depression, hospitalization,  hustle) | 0.5 |
| Specifies medical history  (example: hypertension, diabetes, hepatitis, pancreatitis …) | 0.5 |
| Request weight and height | 0.5 |
| Advice on alcohol consumption and biological assessment  Regarding alcohol consumption:  - Advise to reduce ≤ 3 glasses / day in men  or ≤ 2 glasses / day in women | 0.5 |
| Proposes / evokes help with weaning  (At least one item among: addictology consultation, group of  support, psychotherapy, medication) | 0.5 |
| Explain why decrease  (risk of chronic hepatitis, cirrhosis, pancreatitis, etc.) | 1 |
| Proposes / evokes help with weaning  (At least one item among: addictology consultation, group of  support, psychotherapy, medication) | 1 |
| Advises to reduce paracetamol  (or informs about hepatic risk) | 0.5 |
| Informs that biological abnormalities:  o May be linked to chronic alcohol consumption  o May be linked to other pathologies that need to be explored  (An example cited among: looking for diabetes, metabolic syndrome,  chronic viral hepatitis, nutritional deficiency, liver ultrasound) | 0.5 |
| Asks if the patient has any other questions | 0.5 |
| General attitude (listening, posture, looks in the eyes) | 0.5 |

STATION 3 evaluation grid

| Items | Coefficient |
| --- | --- |
| Looks for a contagion of chickenpox | 1 |
| Looks for a personal history of chickenpox | 1 |
| Asks for the topography of skin lesions | 1 |
| Asks for the evolution of skin lesions | 0.5 |
| Looks for the presence of pruritus | 0.5 |
| Searches for given antipyretic treatments | 0.5 |
| Searches for taking NSAIDs and/or aspirin | 0.5 |
| Asks for history of chickenpox in parents | 1 |
| Chickenpox | 1 |
| Viral infection | 0.5 |
| Explains that there is no need for more exams | 0.5 |
| Spontaneous or usually mild recovery | 0.5 |
| Possible sequelae: prolonged skin scars | 0.5 |
| Antiseptic solution | 1 |
| Antihistamine | 1 |
| Paracetamol | 1 |
| Contraindication to NSAIDs | 1 |
|  |  |
| Contraindication to aspirin | 1 |
| Hygiene: Nails clean and cut short | 0.5 |
| Advices the parent to monitor/consult again if appearance of lesions evoking a  inflammatory plaque superinfection (or pus or abscess) | 1 |
| Advices the parent monitor/reconsult if fever worsens | 0.5 |
| Advices the parent monitor/reconsult in case of balance disorder | 0.5 |
| Advices the parent monitor/consult again in case of worsening general condition | 1 |
| Spontaneously seeks contact with a person at risk (woman  pregnant or immunocompromised person) | 0.5 |
| Specifies the need to avoid contact with pregnant women or  immunocompromised person | 0.5 |
| No compulsory eviction from school | 0.5 |
| Introduces him, her their selves | 0.5 |
| Uses simple and clear language | 0.5 |
|  |  |
|  |  |
|  |  |
|  |  |
|  |  |
|  |  |
